# Comparative effects of pea protein vs. dairy proteins (α-lactalbumin and casein) on aminoacidemia, exercise performance, gut microbiota, hormonal responses, neurocognitive function, mood, and metabolic–inflammatory responses in older adults

**DOI:** 10.3389/fnut.2026.1871477

**Published:** 2026-08-27

**Authors:** Shuai Hao, Fulong Jin, Xiaowei Lei, Yichen He, Yin Liu

**Affiliations:** 1 School of Physical Education, Shaoxing University, Shaoxing, Zhejiang, China; 2 Daegu University, Gyeongsan-si, Gyeongsangbuk-do, Republic of Korea; 3 Sports Department, Chang'an University, Xi'an, Shanxi, China; 4 Xijing Hospital, Air Force Medical University, Xi'an, Shanxi, China

**Keywords:** aminoacidemia, casein, gut microbiota, muscle protein synthesis, neurocognitive function, older adults, pea protein, α-lactalbumin

## Abstract

This narrative review comprehensively contrasts the physiological impacts of plant-based protein (focusing on pea protein isolates and matrices) vs. dairy-derived proteins (including intact casein and whey protein variants, emphasizing the rapid-digesting α-lactalbumin fraction). We establish a structured framework to separately report evidence from preclinical animal models and clinical human trials, evaluating acute (< 24 h) and chronic (>7 days) horizons across three domains: (i) neurocognitive function/mood, (ii) gut microbiota/hormonal responses, and (iii) aminoacidemia/metabolic–inflammatory responses in aging cohorts. Synthesized data reveal that preclinical animal models favor pea protein for altering gut microbiota profiles and elevating short-chain fatty acids, while dairy fractions, particularly α-lactalbumin, optimizing hypothalamic serotonin synthesis and neurocognitive performance via high tryptophan bioavailability. In clinical human trials, acute postprandial metrics heavily favor dairy fractions, inducing rapid leucine spikes and superior peripheral mTORC1 activation compared to pea protein. However, chronic long-term trials indicate that when absolute daily protein and essential amino acid requirements are met, often via leucine fortification or strategic plant–animal blending, discrepancies in muscle protein synthesis and physical performance between pea and dairy proteins are minimized. Ultimately, long-term functional adaptations depend primarily on total daily amino acid adequacy rather than protein origin. Due to a heavy reliance on male-skewed cohorts and a scarcity of direct head-to-head clinical trials comparing pea protein with pure α-lactalbumin fractions, caution must be exercised before making rigid clinical policy decisions for diverse, frail geriatric populations.

## 1 Introduction

Aging is characterized by a progressive deterioration across various physiological systems, particularly in terms of skeletal muscle mass, metabolic regulation, and cognitive functionality (1, 2). This multifaceted decline has profound implications for functional autonomy, overall quality of life, and morbidity among the elderly population (3, 4). One of the most conspicuous manifestations of aging is the insidious reduction of skeletal muscle mass and strength, a phenomenon referred to as sarcopenia, which is intricately associated with frailty, an elevated risk of falls, and metabolic dysregulation (5, 6). Simultaneously, age-associated deficits in glucose metabolism, heightened low-grade inflammation, and cognitive decline further complicate health outcomes within this demographic (7, 8). These interrelated transformations underscore the imperative for holistic nutritional interventions aimed at enhancing muscle, metabolic, and neurological health concurrently. A fundamental mechanism contributing to the diminution of muscle mass with advancing age is anabolic resistance, which is characterized by a diminished muscle protein synthesis (MPS) response to anabolic stimuli, such as the intake of dietary protein and engagement in physical activity (9). Elderly individuals necessitate an augmented intake of protein or protein sources characterized by enhanced anabolic properties to surmount this physiological resistance (10, 11). The caliber of dietary protein, encompassing its amino acid composition, digestibility, and absorption kinetics, is paramount in fostering MPS (12). Specifically, essential amino acids (EAAs), with a particular emphasis on leucine, are instrumental in the activation of the mechanistic target of rapamycin (mTOR) pathway, which serves as a principal regulator of MPS (13). Historically, proteins derived from animals, such as those found in dairy, have been regarded as superior to their plant-based counterparts due to their elevated EAA content and more advantageous digestibility profiles (14, 15). Nonetheless, the burgeoning interest in sustainable and plant-based dietary patterns has engendered an increasing emphasis on plant-derived protein sources, such as pea protein, and their prospective role in promoting healthy aging (16, 17). Therefore, comprehending the relative efficacy of plant vs. animal proteins across various physiological domains is of substantial significance.

To counteract these multi-system declines, targeted nutritional strategies have increasingly focused on comparing plant-derived alternatives with traditional animal-derived options, beginning with their distinct nutritional profiles.

### 1.1 Pea protein

Pea protein is a protein derived from plant sources that exhibits moderate digestibility (approximately 80%−90%) and is distinguished by a balanced yet incomplete profile of EAAs. It is particularly rich in arginine and lysine; however, it exhibits limitations in sulfur-containing amino acids, notably methionine and cysteine, which may restrict its anabolic efficacy when compared to proteins sourced from animals. The leucine concentration in pea protein is moderate (approximately 7%−8%), which falls short of the levels found in numerous dairy proteins, potentially impacting the efficacy of MPS stimulation in older populations. Nevertheless, pea protein demonstrates relatively advantageous gastric emptying and moderate digestive kinetics, resulting in a sustained, albeit less pronounced, elevation in amino acid levels. Research suggests that although pea protein can contribute to muscle preservation, elevated dosages may be necessary to achieve an anabolic response comparable to that elicited by whey or casein, attributable to its amino acid deficiencies (18, 19).

### 1.2 Dairy proteins

Dairy-derived proteins encompass casein and whey components, particularly the whey fraction including α-lactalbumin. Both of these demonstrate elevated digestibility levels exceeding 95% and encompass comprehensive essential amino acid profiles. Notably, α-lactalbumin is abundant in tryptophan, a biochemical precursor to serotonin, which may exert an influence on mood and neurocognitive capabilities in the elderly population. It undergoes rapid digestion, resulting in a pronounced increase in plasma amino acids and insulin levels, thus significantly enhancing MPS. Conversely, casein is characterized as a slow-digesting protein that forms a gel-like clot in the gastric environment, resulting in an extended release of amino acids and enduring anti-catabolic benefits. This phenomenon of “slow” aminoacidemia proves advantageous for the retention of protein overnight and the preservation of muscle mass. Collectively, dairy proteins furnish both immediate and prolonged anabolic signaling, rendering them exceptionally efficacious in promoting muscle health and metabolic regulation among aging demographics (20, 21).

### 1.3 Traditional protein quality metrics vs. functional performance indicators

To systematically categorize these differences in digestibility and amino acid profiles, the nutritional value of plant and animal proteins has traditionally relied on standardized scoring indices. Evaluating the nutritional value of plant and animal proteins traditionally relies on standardized scoring indices, specifically the Protein Digestibility-Corrected Amino Acid Score (PDCAAS) and the newer, more accurate Digestible Indispensable Amino Acid Score (DIAAS) (22, 23). Dairy proteins consistently exhibit optimal values under these systems; intact casein and whey-derived fractions like α-lactalbumin routinely achieve DIAAS scores exceeding 1.00 (or 100%), reflecting their high essential amino acid density and superior ileal digestibility (23). Conversely, plant-derived alternatives like pea protein isolate exhibit slightly depressed scores, typically ranging between 0.75 and 0.90, primarily limited by a lower concentration of sulfur-containing amino acids such as methionine and cysteine (22).

While PDCAAS and DIAAS remain indispensable for population-level dietary planning and safeguarding against basic deficiency states, a growing body of evidence suggests they do not tightly correlate with acute, real-time physiological performance outcomes in older populations (24, 25). These conventional metrics assess overall amino acid disappearance and fecal/ileal retention rather than the specific metabolic kinetics required to stimulate peripheral target tissues (23, 25).

Crucially, traditional scores fail to capture the postprandial blood amino acid appearance rate (aminoacidemia) and the subsequent magnitude of the intramuscular leucine trigger (24). In older adults characterized by anabolic resistance, overcoming the threshold for muscle protein synthesis (MPS) demands rapid, transient spikes in arterial leucine concentrations to stimulate mammalian target of rapamycin complex 1 (mTORC1) signaling (24). Consequently, two protein sources with vastly different digestion velocities can yield identical DIAAS scores while exerting radically divergent acute impacts on fractional synthetic rates (24, 25). Relying strictly on traditional protein quality metrics can mask these crucial kinetic variations, underscoring the need to balance static scores with dynamic, functional performance parameters when designing clinical nutrition strategies for aging cohorts (24, 25).

To bridge the gap between structural amino acid presence and actual functional metabolic outcomes, recent food science frameworks have introduced the Branched-Chain Amino Acid Score (BCAAS score) as a target indicator for muscle remodeling (26). While traditional metrics like PDCAAS show a weak correlation with acute postprandial muscle protein synthesis (*r* < 0.44), the BCAAS score demonstrates a substantially higher correlation coefficient (*r* = 0.74) with MPS outcomes (26). Using whey protein as the reference baseline (scaled at 100), the BCAAS score exposes distinct variations among protein types: pea protein isolate scores lowest at 44.36, intact casein scales at 74, whole milk at 78.85, and α-lactalbumin reaches an optimal score of 115 due to its dense indispensable profile and 95% digestibility kinetics (26, 27). An acknowledged limitation of the current BCAAS framework is that its evidentiary validation has been primarily established in younger populations (26). Nevertheless, it provides an invaluable predictive benchmark for the food industry when engineering functional recovery foods optimized for geriatric demographics. In practical application, the relatively low baseline BCAAS score of pea protein (44.36) does not represent an immutable restriction; rather, it highlights a clear bioengineering opportunity. Targeted free leucine supplementation or precise blending with dairy fractions can successfully adjust the total amino acid architecture, increasing the composite BCAAS score of plant-based matrices to match or exceed dairy standards, thereby enhancing its clinical utility in treating age-related muscle wasting (26, 27). However, evaluating protein quality solely through the lens of acute kinetic scoring frameworks offers an incomplete picture of geriatric health. Shifting the clinical focus toward whole-food matrices reveals broader physiological impacts that extend across the gut–brain–muscle axis.

To facilitate a comprehensive biochemical comparison, the typical amino acid compositions of pea protein isolate, intact casein, and α-lactalbumin are summarized below (Table 1).

**Table 1:** Representative amino acid profiles of pea protein isolate, intact casein, and α-lactalbumin (g/100 g of protein) (19, 27).

Amino acid	Pea protein isolate	Intact casein	α-lactalbumin
Essential amino acids (EAAs)			
Leucine	7.8	9.2	11.4
Isoleucine	4.5	4.9	6.2
Valine	4.8	6.1	4.4
Lysine	7.2	7.6	10.5
Methionine	0.9	2.6	0.9
Cysteine	1.0	0.4	5.8
Phenylalanine	5.3	4.8	4.1
Tyrosine	3.7	5.1	4.8
Threonine	3.6	4.1	4.7
Tryptophan	0.9	1.3	5.6
Histidine	2.5	2.6	2.7
Non-essential amino acids (NEAAs)			
Arginine	8.4	3.6	1.1
Alanine	4.2	2.9	3.1
Aspartic acid/asparagine	11.5	6.8	16.2
Glutamic acid/glutamine	16.8	21.4	12.9
Glycine	4.0	1.8	1.9
Proline	4.3	10.3	1.3
Serine	5.1	5.6	4.2

While a substantial body of literature addresses individual aspects of protein nutrition, existing comparative reviews frequently isolate skeletal muscle adaptations, gut microbiota configurations, or cognitive health into independent discussions. There remains a distinct research gap concerning a system-level integration that connects these disparate physiological axes specifically for the aging demographic. To address these systemic complexities and bridge the gap between isolated datasets, this narrative review provides a highly structured, dual-matrix evaluation of plant-based protein (focusing on pea protein isolates and matrices) vs. dairy-derived proteins (specifically intact casein and the rapid-digesting α-lactalbumin whey fraction). The structural novelty of our approach lies in its organized, dual-matrix evaluation: we explicitly separate the evidence derived from preclinical animal models from clinical human trials, while concurrently categorizing both acute (postprandial) and chronic (long-term) exposure horizons across three unified functional domains: (i) neurocognitive function and mood regulation, (ii) gut microbiota and hormonal responses, and (iii) aminoacidemia and metabolic–inflammatory markers. By providing this consistent, multi-system perspective, this work seeks to identify evidence gaps, which can highlight conflicting findings and propose a structured roadmap for targeted functional food engineering in geriatric care.

## 2 Methods

To map the physiological landscape connecting plant and dairy protein nutrition, a comprehensive literature search was executed across electronic databases (PubMed, Scopus, and Web of Science) for articles published up to 2026. To address the inherent differences in physiology, experimental protocols, exposure timelines, and measurement tools, literature eligibility was split into two separate selection streams.

### 2.1 Eligibility parameters for clinical human trials

- **Inclusion criteria:**
  ° **Population:** Randomized controlled trials (RCTs), cross-over designs, and clinical cohorts evaluating healthy, sarcopenic, or frail older adults (mean age 60 years).
  ° **Interventions:** Direct administration of pea protein (isolates, concentrates, or blends) compared head-to-head or against independent controls utilizing dairy proteins (intact casein, whey isolates, or α-lactalbumin fractions).
  ° **Time Horizons:** Categorized strictly into *Acute* (immediate postprandial changes assessed over < 24 h) and *Chronic* (long-term dietary changes evaluated over >7 days).
  ° **Outcomes:** Validated human metrics including arterial aminoacidemia, fractional synthetic rate (FSR) of muscle, blood metabolic/inflammatory biomarkers, gut microbiota operational taxonomic units (OTUs), plasma gastrointestinal hormones, and validated mood/cognitive assessment scores.
- **Exclusion Criteria:** Studies examining multi-ingredient supplements containing non-protein anabolic agents (e.g., creatine, anabolic steroids), or cohorts exclusively comprising young or middle-aged adults (< 60 years).

### 2.2 Selection parameters for preclinical animal models

- **Inclusion criteria:**
  ° **Models:** *In vivo* rodent models (mice or rats) configured as aged phenotypes (typically >18 months) or established metabolic acceleration models.
  ° **Interventions:** Controlled dietary intake or acute gavage of pea-derived proteins vs. animal-derived dairy protein baselines (casein or particularly the whey fraction including α-lactalbumin).
  ° **Time Horizons:** Defined dynamically based on rodent lifespans, where acute exposure evaluates immediate molecular signaling (< 24 h) and chronic exposure captures systemic phenotypic changes (>4 weeks).
  ° **Outcomes:** Preclinical mechanistic endpoints, including muscle protein synthesis signaling paths (mTORC1/p70S6K phosphorylation), tissue-isolated inflammatory cytokines, direct cecal/fecal short-chain fatty acid (SCFA) quantification, intestinal histomorphometry, and rodent behavioral tests (e.g., Morris water maze or open field tests for neurocognition and mood).
- **Exclusion Criteria:** *In vitro* cell culture models lacking corresponding *in vivo* animal validation, non-mammalian models, and studies utilizing protein-deficient starvation protocols that mask typical age-related metabolic parameters.

## 3 Summary of evidence

### 3.1 Evidence from animal studies

#### 3.1.1 Acute effect

In an acute (< 24 h) animal model investigation aimed to ascertain whether plant-derived protein (specifically a pea-soy amalgamation, referred to as PS, an isolate/blend) can replicate the anabolic effects observed with whole whey protein in male geriatric mice. In a cohort of C57BL/6J mice aged 25 months, a regimen of oral gavage was employed, administering either 70 mg of whey (W), PS, leucine-enhanced PS (designated PS + L), or a control solution of water. The assessment of MPS was conducted utilizing the SUnSET methodology. Notably, MPS exhibited a statistically significant increase in response to both W and PS + L (*P* < 0.003), whereas PS alone did not elicit a similar response. The signaling protein 4EBP1 demonstrated elevated levels across all protein treatment groups in comparison to the control (*P* < 0.0002). Both W and PS + L effectively augmented plasma and muscle leucine concentrations, a finding corroborated through dried blood spot analysis. Collectively, these results suggest that the fortification of plant protein blends with leucine facilitates the stimulation of MPS to a degree comparable with whey protein, thereby underscoring leucine's pivotal role as a catalyst for anabolic responses in the aging process (28).

In addition, another acute (< 24 h) animal model research assessed the impact of a hybrid blend protein composition (P4: comprising whey, casein, pea, and soy) vs. whole protein (whey and casein) on the process of MPS in male aged C57BL/6J mice, specifically those that are 25 months old. After a period of overnight fasting, the mice were administered either whey, P4, casein, or a control of water. The results demonstrated a 1.6-fold increase in MPS with whey (*P* = 0.006) and a 1.5-fold increase with P4 (*P* = 0.008) in comparison to the fasted controls, whereas casein did not exhibit any significant effect. This finding was corroborated by elevated phosphorylated to total 4E-BP1 ratios in the whey (*P* = 0.012) and P4 (*P* = 0.001) groups, with no observed alterations in mTOR or p70S6K signaling pathways. Furthermore, the intramuscular levels of leucine were found to be lower in the P4 group (0.71 μmol/g) compared to the whey group (0.97 μmol/g; *P* = 0.0007). Nevertheless, P4 significantly enhanced the concentrations of circulating amino acids 10 min following the consumption of food (29) (Table 2).

**Table 2:** Acute effects of pea protein vs. dairy protein on muscle protein metabolism, amino acid availability, and functional outcomes in aging: a summary of preclinical evidence of animal studies.

Age/gender/ health status	Time horizon	Dose & duration	Form of protein/type of protein	Key outcomes	Findings	References
25-month-old/Males/Healthy aging (Mice)	Acute (< 24 h)	Single dose 70 mg; 60 min follow-up	Whole protein (Whey) vs. Isolate/Blends (Pea-soy blend) ± Leucine	MPS, mTOR signaling, amino acids	MPS: ↑ whey = ↑ pea+leucine > ↔ pea • mTOR signaling: ↑ all protein groups vs. control • Plasma/muscle leucine: ↑ whey = ↑ pea+leucine > pea	(28)
25-month-old/Males/Fasted healthy aging (Mice)	Acute (< 24 h)	Overnight fast + single oral gavage; outcomes ~60 min	Whole protein (Whey, Casein) vs. Hybrid blend (P4: whey + casein + pea + soy)	MPS, mTOR signaling, amino acids	MPS: ↑ whey = ↑ P4 > ↔ casein • 4E-BP1 phosphorylation: ↑ whey = ↑ P4 • mTOR/p70S6K: ↔ • Intramuscular leucine: ↓ P4 < whey • Postprandial AA (early): ↑ P4 vs. fasted	(29)

#### 3.1.2 Chronic effects

A chronic (>7 days) animal model study elucidated the impacts of whole pea protein, both in the presence and absence of inulin, on muscular health in male senescent rats aged 20 months over a duration of 16 weeks. The subjects were allocated to either a diet consisting solely of pea protein (PEA) or a diet comprising pea protein in conjunction with inulin (PEA + INU). Both cohorts exhibited significant postprandial activation of p70S6K phosphorylation, thereby indicating the stimulation of anabolic signaling pathways. Nonetheless, the PEA + INU cohort exhibited a superior preservation of muscle mass over the duration of the study, alongside a diminished expression of MuRF1, which serves as a biomarker for muscle protein catabolism. Furthermore, the supplementation of inulin was associated with an enhancement in PGC-1α expression and an increase in mitochondrial enzyme activity within the muscular tissue (30).

An animal model investigation determined the nutritional efficacy of isolate pea protein in comparison to whole protein casein and whey in male senescent rats (20 months of age) over a chronic period of 16 weeks. A cohort of 30 male Wistar rats was administered isocaloric and isonitrogenous diets that included casein, whey, or PPI. The findings revealed no statistically significant differences among the groups in terms of nitrogen balance, true digestibility, or net protein utilization. Furthermore, parameters such as body composition, tissue weight, MPS and degradation, mitochondrial function, inflammation, and insulin resistance exhibited comparability across all protein sources. These results suggest that pea protein is assimilated with equal efficacy as casein and whey in aged rats. Consequently, pea protein serves as a plausible alternative to animal-derived proteins, thereby facilitating dietary diversification while maintaining metabolic and muscular health in the aging population (31).

A chronic animal model research examined the nutritional composition of whole protein wheat pasta augmented with leguminous flours (specifically faba bean, lentil, or split pea) in relation to dairy proteins (casein or soluble milk proteins, SMP) in male geriatric rats over a duration of 6 weeks. A cohort of 43 aged rats ingested isocaloric and isoproteic diets that included either legume-enriched pasta or milk-derived proteins (namely casein or soluble milk proteins, SMP). The digestibility of proteins exhibited a marginally higher degree for dairy proteins (ranging from 5–14%) in contrast to the legume-based pasta. Nevertheless, metrics such as net protein utilization, skeletal MPS, and overall muscle mass were found to be equivalent between the legume-enriched pasta and casein, although SMP demonstrated superior anabolic responses. The rate of muscle protein accretion appeared to be consistent across both legume and dairy protein groups (32).

An animal model investigation conducted a comparative analysis of the effects of whole protein casein (characterized by slow digestion), whey, and soluble milk protein (noted for rapid digestion) in conjunction with or without low-intensity treadmill exercise in male senescent Wistar rats (aged 17–19 months) over a chronic (>7 days) duration of 2 months. In the absence of exercise, the administration of protein supplements yielded negligible effects, evidenced by modest increases in muscle mass within the casein cohort and slight enhancements in gait observed with the soluble milk protein. Conversely, when these protein types were administered in conjunction with physical activity, the soluble milk protein exhibited the most significant advantages, markedly enhancing locomotor activity, gait metrics, and functional performance in comparison to whey or casein, notwithstanding the absence of variations in muscle mass or forelimb strength. These results imply that the type of protein as well as the timing of its administration relative to exercise may exert a greater influence on functional mobility rather than muscle hypertrophy in the aging process. Rapidly digestible milk proteins administered post-activity may more effectively facilitate locomotor function and physical performance in older populations (33).

A 6-month chronic investigation involving both genders of healthy aged rats in an animal model examined the effects of whole protein casein (12% protein) and whey protein (12% and 18%), both in the presence and absence of a blend of antioxidants (chamomile extract, vitamin E, vitamin D). The outcomes assessed encompassed body composition, inflammatory responses, oxidative stress levels, MPS, and proteolytic activity. While the supplementation of antioxidants led to a reduction in inflammatory markers and oxidative stress, it did not yield a statistically significant impact on muscle mass. The sole intervention that demonstrably mitigated the loss of lean mass was an elevation in whey protein intake (18%), which exhibited a tendency to decrease muscle proteolysis yet showed no definitive alterations in MPS. Casein was found to be less efficacious than whey in the preservation of lean mass (34).

In addition, a chronic animal model research investigated the efficacy of pancreatic extract (PE) vs. whole protein casein supplementation in facilitating the recovery from endotoxin-induced catabolic stress in both genders of aged rats (24 months of age). Subsequent to the administration of lipopolysaccharide, the subjects exhibited notable weight reduction (~7.6%). Throughout a 7-day recovery phase, the rats were administered either PE or isonitrogenous casein, while ensuring that their food intake remained consistent. The findings revealed that in fast-twitch (white) muscle, casein supplementation was ineffective in mitigating protein depletion and resulted in diminished glutamine levels. Conversely, pancreatic extract notably reduced muscle wasting and enhanced intramuscular glutamine concentrations. No significant alterations were noted in slow-twitch muscle across either group (35). Another chronic animal model research examined the anti-aging properties of whole protein/fraction bovine α-lactalbumin in relation to cardiovascular aging through the utilization of both H_2_O_2_-induced cellular senescence models and both genders of aged murine subjects. *In vitro* studies demonstrated that α-lactalbumin effectively attenuated senescence-associated β-galactosidase activity and downregulated critical markers of aging (p16, p21, p53), in addition to mitigating inflammation and oxidative stress. *In vivo* investigations revealed that aged mice administered α-lactalbumin exhibited diminished cardiovascular senescence as assessed by both histological and molecular aging biomarkers. Mechanistically, the protective effects were linked to the upregulation of Sirtuin 1 (SIRT1), a pivotal regulator of cellular stress resistance and the aging process (36) (Table 3).

**Table 3:** Chronic effects of pea protein vs. dairy protein on muscle protein metabolism, amino acid availability, and functional outcomes in aging: a summary of preclinical evidence of animal studies.

Age/gender/ health status	Time horizon	Dose & duration	Form of protein/type of protein	Key outcomes	Findings	References
20-month-old/Males/Healthy aging (Rats)	Chronic (>7 days)	16 weeks dietary intervention	Whole protein/Pea protein diet vs. Pea + Inulin	Muscle mass, mitochondrial function, protein signaling	Muscle mass: ↑ PEA+INU > ↑ PEA • MuRF1 (atrophy marker): ↓ PEA+INU • P70S6K phosphorylation: ↑ both groups • PGC-1α expression: ↑ PEA+INU • Mitochondrial enzyme activity: ↑ PEA+INU	(30)
20-month-old/Males/Healthy aging (Wistar rats)	Chronic (>7 days)	16 weeks isoproteic/isocaloric diet	Isolate (Pea protein isolate) vs. Whole protein (Casein, Whey)	Protein retention, MPS, metabolism, inflammation	Nitrogen balance: ↔ casein = WHEY = PEA • MPS: ↔ • Muscle protein degradation: ↔ • Mitochondrial activity: ↔ • Insulin resistance: ↔ • Overall protein efficiency: ↑ PEA = milk proteins	(31)
Old/Males/Healthy aging (Rats)	Chronic (>7 days)	6 weeks isoproteic/isocaloric diet	Whole protein/Wheat–legume pasta blends vs. Casein vs. Soluble milk protein	Protein digestibility, MPS, muscle mass	Digestibility: ↓ legume pasta < milk proteins • MPS: ↑ SMP > ↔ casein = legume pasta • Whole-body protein retention: ↔ casein = legume pasta • Muscle mass: ↔ all groups (similar accretion)	(32)
17–19 months/Males/ Healthy aging (Wistar rats)	Chronic (>7 days)	2 months; protein 5 × /week + low-intensity treadmill	Whole protein/Casein vs. Whey vs. Soluble milk protein ± Activity	Locomotion, gait, muscle function	Muscle mass: ↑ casein (sedentary) • Stride frequency: ↑ soluble milk protein • With activity: ↑ soluble milk protein > casein = whey • Locomotor function: ↑ soluble milk protein + activity • Muscle mass/strength (active): ↔	(33)
Old/Both genders/Healthy aging (Rats)	Chronic (>7 days)	6 months; 12–18% protein diets ± antioxidants	Whole protein/Casein vs. Whey (12% vs. 18%) ± Antioxidants	Lean mass, inflammation, MPS, proteolysis	Lean body mass: ↑ whey (18% > 12% > casein) • Muscle protein synthesis: ↔ • Muscle proteolysis: ↓ whey (trend) • Inflammation/oxidative stress: ↓ antioxidant groups (no muscle effect) • Overall sarcopenia prevention: ↑ whey intake	(34)
Aged/Both Genders/ Cardiovascular aging (Mice)	Chronic (>7 days)	Continuous dietary supplementation exposure	Whole protein/Fraction/ Bovine α-lactalbumin	Senescence markers, inflammation, oxidative stress, Sirt1	Senescence markers (p16/p21/p53, SA-β-gal): ↓ • Inflammation: ↓ • Oxidative stress: ↓ • Sirt1 signaling: ↑ • Overall cardiovascular aging: ↓	(36)
24 months/Both Genders/LPS-induced endotoxemia (Rats)	Chronic (>7 days)	7 days post-LPS recovery framework	NA (Extract) vs. Whole protein (Casein)/Pancreatic extract vs. Casein	Muscle protein content, glutamine pool, catabolic status	Body weight: ↓ LPS-induced loss • Fast-twitch muscle protein: ↓ casein group • Glutamine content: ↑ PE group • Muscle wasting: ↓ PE > casein • Slow muscle: ↔ • Overall nutritional recovery: ↑ PE	(35)

### 3.2 Evidence from human studies

#### 3.2.1 Acute effects

In a single-blind randomized human model investigation involving both genders of middle-aged to older adults, the acute (< 24 h) implications of incorporating low-dose protein (~0.13 g/kg body mass) into a low-protein breakfast (~0.07 g/kg body mass) were evaluated through the utilization of concentrate whey protein (WPC) and isolate pea protein (PPI). Venous blood specimens were obtained over a duration of 180 min postprandially to examine amino acid levels and the concentrations of appetite-regulating hormones. Plasma concentrations of total and EAAs exhibited a statistically significant increase over time (*P* < 0.05), with no notable discrepancies between the groups regarding overall availability (iAUC). Nevertheless, plasma leucine concentrations demonstrated significantly higher peak levels (*P* = 0.032) and iAUC (*P* = 0.012) within the WPC cohort in comparison to the PPI group. The levels of appetite-related hormones (ghrelin, GLP-1) and subjective appetite assessments exhibited temporal changes (*P* < 0.05), with no significant differences observed between the groups (37).

A randomized, single-blind, crossover human model investigation evaluated the comparative acute impacts of isolate whey protein (WPI) and isolate pea protein (PPI) on appetite and metabolic processes in younger (25.2 ± 2.8 years, *n* = 15) and older male subjects (67.7 ± 4.5 years, *n* = 15). Participants ingested a breakfast consisting of 40 grams of protein, and physiological responses were assessed over a duration of 4 h. Appetite was significantly modulated by temporal factors, age, and the protein source (*P* < 0.05); nevertheless, no statistically significant disparities were detected between WPI and PPI with respect to appetite-regulating hormones, caloric intake, energy expenditure, or substrate oxidation. Upon controlling for body weight, older male participants exhibited reduced energy expenditure (*P* < 0.05) and diminished fat oxidation (*P* < 0.001) when contrasted with their younger counterparts. In summary, both sources of protein demonstrated comparable influences on appetite modulation and metabolic responses, irrespective of the age demographic (38).

Another randomized, double-blind, crossover human model study aimed to elucidate acute postprandial amino acid responses to various whole protein sources in both genders of young adults (22 ± 1 years, *n* = 12) and older adults (69 ± 2 years, *n* = 10). Participants ingested 30 g of protein derived from milk, pea, lupin, mycoprotein, spirulina, or chlorella, with subsequent blood samples collected over a duration of 5 h. The ingestion of protein significantly elevated plasma concentrations of total and EAAs (*P* < 0.001), revealing pronounced differences among the protein sources (*P* < 0.001), while age was found to further influence these responses (*P* < 0.001). Notably, pea protein and spirulina elicited the highest peak concentrations of amino acids, in contrast to chlorella, which exhibited the lowest levels. The overall availability of amino acids was maximized for pea, spirulina, and mycoprotein, whereas it was minimized for chlorella, with no consistent age-related differences observed in peak concentrations (39).

Another human model research assessed acute postprandial amino acid responses to various whole protein and blends among both genders of healthy older adults (72.3 ± 3.4 years, BMI 25.3 ± 2.9 kg/m^2^). Participants ingested 20 g of either pea protein, milk protein, micellar casein, or a 60/40 casein–pea protein blend, with plasma amino acids evaluated over a duration of 5 h. The casein–pea protein blend significantly enhanced the availability of total and EAAs (AUC and peak height) in comparison to pea protein alone, particularly with regard to leucine and methionine, while concurrently sustaining elevated levels of arginine. The amino acid response elicited by the blend was found to be intermediate between that of its constituent proteins, thereby indicating potential complementary effects (40).

A randomized, single-blind crossover human model investigation evaluated acute postprandial amino acid responses to whole protein and blends (specifically plant protein composites/pea-derived mixtures) in contrast to whey protein supplemented with fiber in a cohort of both genders of healthy older adults (≥65 years, *n* = 9). All meals were meticulously standardized for EAAs and leucine content. Blood specimens were procured over a duration of 3 h subsequent to ingestion. The plant protein–fiber formulations elicited lower peak levels of leucine, BCAAs, and indispensable amino acids relative to whey, although the overall AUC remained comparable. The bioavailability of methionine, cysteine, and threonine was similarly diminished following the consumption of plant-based proteins. Whey protein was associated with heightened postprandial insulin responses compared to plant protein composites. These findings elucidate that the kinetics of amino acid appearance and associated hormonal responses are significantly influenced by the source of protein in older adults, implying a potentially reduced anabolic signaling with plant-based formulations despite equivalent leucine concentrations (41).

In another randomized controlled tracer human model investigation, the acute impact of whole protein sources on protein digestion and MPS was evaluated in elderly males (74 ± 1 years, *n* = 48). Stable isotope methodologies were employed to analyze the kinetics of amino acid appearance as well as the muscle FSR. The findings indicated that whey and casein hydrolysate elicited a more rapid peak plasma phenylalanine appearance in comparison to intact casein (*P* < 0.05). Notably, whey was associated with a significantly elevated rate of MPS (0.15 ± 0.02%/h) relative to casein (0.08 ± 0.01%/h; *P* < 0.01) and hydrolysate (0.10 ± 0.01%/h; *P* < 0.05). Furthermore, a robust correlation was identified between peak leucine concentrations and MPS (*r* = 0.66, *P* < 0.01) (42).

A human model study determined the acute metabolism of tryptophan (TRP) and associated mood responses among both genders of older adults diagnosed with mild cognitive impairment (MCI; *n* = 32) compared to matched control subjects (*n* = 26), with a specific subset consuming a meal rich in whole protein/fraction α-lactalbumin (ALAC). Initial findings revealed significantly elevated levels of plasma anthranilic acid in individuals with MCI (*P* = 0.015). Subsequent to the intake of ALAC, both cohorts demonstrated notable increases in TRP and metabolites associated with the kynurenine pathway (including kynurenine, 3-hydroxykynurenine, kynurenic acid, picolinic acid, and anthranilic acid; all *P* < 0.001), while serotonin levels did not exhibit significant variation. The availability of TRP, as determined by the TRP/large neutral amino acid ratio, experienced a substantial increase in both groups. Participants with MCI exhibited a pronounced elevation in picolinic acid concentrations (*P* < 0.001). Despite these metabolic alterations, no immediate enhancements in mood, as measured by the Profile of Mood States, were detected (43) (Table 2).

Furthermore, a separate randomized controlled trial investigated the acute effects of hydrolysate whey, whole protein caseinate, and carbohydrate on MPS in an older male population (69 ± 1 years, *n* = 27) utilizing a unilateral resistance exercise paradigm. Participants ingested 0.45 g/kg of lean body mass for each respective intervention, and myofibrillar FSR was assessed at baseline, following feeding, and after exercise. Both whey protein and caseinate significantly elevated fed-rest and fed-exercise FSR in comparison to basal levels (*P* < 0.001), whereas carbohydrate yielded comparatively modest enhancements. Nonetheless, no statistically significant disparities were detected among the protein types at any temporal measurement. In a similar vein, p70S6K activity, along with postprandial amino acid and insulin responses, exhibited no significant variances across the different protein groups. Resistance exercise did not further amplify MPS within the 0–3 h postprandial timeframe (44).

A double-blind randomized controlled human model trial examined the acute immediate effects of hydrolysate/peptide casein (TMP), whole protein intact casein (TMC), and placebo (TMF) on cognitive performance and neurophysiological activity among both genders of cognitively healthy older adults (*n* = 47). Participants ingested the supplements 30 min prior to the assessment, and executive function was evaluated through a task-switching reaction time paradigm in conjunction with fMRI imaging. No statistically significant differences were detected among the groups with respect to reaction time, accuracy, or switch cost. Nonetheless, fMRI findings indicated notable alterations in neural activity within the TMP group, which encompassed reduced activation in the supplementary motor cortex and posterior cingulate gyrus, alongside heightened activation in the amygdala relative to the control group. Additionally, subjective mood ratings (VAS scores) were significantly elevated in the TMP group, with no adverse effects documented (45).

A separate single-blind, randomized crossover human model investigation assessed the acute immediate impacts of whole protein pre-sleep consumption of casein protein vs. maltodextrin and water on next-morning appetite and metabolic responses in both genders of an elderly population (71.3 ± 4.2 years, *n* = 12). Following a standardized evening meal, participants ingested either a 40 g protein beverage, an isocaloric maltodextrin solution, or water prior to sleep. The subsequent morning, appetite, energy intake during breakfast, resting metabolic rate, respiratory exchange ratio, and plasma concentrations of hormones (ghrelin, leptin, insulin, glucose) were evaluated. No statistically significant differences were detected among the conditions in terms of appetite, energy intake, metabolic rate, or hormonal responses. These results indicate that the consumption of protein prior to sleep does not adversely affect next-morning appetite or metabolic processes, implying that it may serve as an effective strategy to enhance total daily protein consumption in older adults without detrimentally influencing subsequent food intake or metabolic homeostasis (46) (Table 4).

**Table 4:** Acute effects of pea protein vs. dairy protein on muscle protein metabolism, amino acid availability, and functional outcomes in aging: a summary of clinical evidence of human studies.

Age/gender/ health status	Time horizon	Dose & duration	Form of protein/type of protein	Key outcomes	Findings	References
Middle-to-older aged/both genders/healthy	Acute (< 24 h)	Single breakfast ingestion; 180 min follow-up	Concentrate (whey) vs. Isolate (pea protein isolate)	Plasma amino acids, ghrelin, glp-1, appetite ratings	Leucine response: ↑ whey > pea • Total amino acids: ↔ • Appetite hormones: ↔ • Appetite ratings: ↔	(37)
Young (25 y) vs. older (68 y)/males/healthy	Acute (< 24 h)	40 g protein meal; 4 h postprandial crossover	Isolate/whey protein isolate vs. Pea protein isolate	Appetite, energy expenditure, fat oxidation	Appetite: ↔ whey vs. pea • Energy expenditure: ↔ protein source • Food intake: ↔ • Fat oxidation: ↓ older vs. young (age effect) • Overall protein effect: ↔ whey = pea	(38)
Young (22 y) vs. older (69 y)/both genders/healthy	Acute (< 24 h)	30 g protein dose; 5 h postprandial crossover	Whole protein/milk vs. Pea vs. Spirulina vs. Chlorella vs. Lupin vs. Mycoprotein	Plasma amino acids, insulin, glucose	Total aa response: ↑ pea = spirulina > other plant proteins • Essential aa: ↑ pea = spirulina > chlorella • Age effect: ↔ (no major difference in magnitude) • Amino acid availability: ↑ pea = spirulina = mycoprotein > chlorella	(39)
~72.3 ± 3.4 y/both genders/healthy	Acute (< 24 h)	20 g protein dose; 5 h postprandial assessment	Whole protein & blends/pea vs. Milk protein vs. Casein vs. Casein–pea blend (60/40)	Plasma amino acids, leucine, methionine auc	Total aa availability: ↑ casein–pea > pea • Leucine: ↑ casein–pea > pea • Methionine: ↑ casein–pea > pea • Arginine: ↔ (preserved in pea component) • Overall response: ↑ blend (intermediate between pea and casein)	(40)
≥65 years/both genders/healthy	Acute (< 24 h)	3 h postprandial crossover; leucine-matched meals	Whole protein & blends/whey protein + fiber vs. Plant protein–fiber blends	Plasma amino acids, insulin response	Leucine appearance: ↓ ppf < wpf • bcaa/iaa: ↓ ppf < wpf • Methionine/cysteine: ↓ ppf < wpf • Insulin: ↓ ppf < wpf • aa availability: ↔ iauc (some measures similar) • Overall anabolic potential: ↓ plant blends < whey	(41)
~74 ± 1 y/males/healthy	Acute (< 24 h)	Single dose 20 g labeled protein tracking	Whole protein & hydrolysate/whey vs. Casein vs. Casein hydrolysate	Muscle protein synthesis (fsr), amino acid kinetics	Mps (fsr): ↑ whey > casein hydrolysate > casein • Plasma leucine peak: ↑ whey = hydrolysate > casein • Protein digestion rate: ↑ whey = hydrolysate > casein • Muscle accretion: ↑ whey strongest • Correlation leucine–mps: ↑ strong positive (*r* = 0.66)	(42)
≥55 y/both genders/mild cognitive impairment & controls	Acute (< 24 h)	Single meal consumption; 3 h postprandial tracking	Whole protein/fraction/ alpha-lactalbumin (trp-rich protein meal)	Plasma trp metabolites, mood (poms), serotonin pathway	Trp, kynurenine pathway metabolites: ↑ (trp, kynurenine, kyna, 3-hk, picolinic acid) • Serotonin: ↔ • trp/lnaa ratio: ↑ • Mood: ↔ • Picolinic acid: ↑ in mci > controls	(43)
~69 ± 1 y/males/healthy	Acute (< 24 h)	Single resistance exercise bout + 0.45 g/kg lbm	Hydrolysate (whey) vs. Whole protein (caseinate) vs. Carbohydrate	Myofibrillar protein synthesis (fsr), p70s6k signaling	Fsr (rested): ↑ protein > cho • fsr (exercise): ↑ protein > cho • Whey vs. casein: ↔ • Resistance exercise effect (0–3 h): ↔ additional benefit • p70s6k: ↔ between proteins	(44)
Elderly/both genders/cognitively healthy	Acute (< 24 h)	Single dose 30 min pre-cognitive assessment	Hydrolysate/peptide (casein peptide) vs. Whole protein (casein)	Cognitive performance, fmri brain activity	Cognitive performance: ↔ • smc & pcg activity: ↓ tmp (casein peptide) • Amygdala activity: ↑ tmp • Mood (vas): ↑ tmp • Overall neural modulation: ↑ casein peptide	(45)
~71.3 ± 4.2 y/both genders/healthy	Acute (< 24 h)	Single 40 g pre-sleep beverage; next-morning tracking	Whole protein/casein protein vs. Maltodextrin vs. Water	Appetite, energy intake, rmr, rer, hormones	Appetite (next morning): ↔ • Energy intake: ↔ • rmr: ↔ • rer: ↔ • Ghrelin, leptin, insulin: ↔ • Overall metabolic impact: ↔ • Protein intake strategy feasibility: ↑ casein (safe, no compensation)	(46)

#### 3.2.2 Chronic effects

A randomized, double-blind human model investigation assessed the chronic impact of isolate protein supplementation on MPS among older males (72 ± 4 years) who were experiencing anabolic resistance. A total of thirty-one subjects adhered to a controlled diet at the recommended dietary allowance (RDA) for a period of 7 days, followed by an eight-day regimen of additional protein supplementation (50 g/day) derived from whey (*n* = 10), pea (*n* = 11), or collagen (*n* = 10). The integrated MPS exhibited a significant increase with whey (1.59 ± 0.11 %/d; *P* < 0.001) and pea protein (1.59 ± 0.14 %/d; *P* < 0.001) in comparison to the RDA (~1.46 %/d), whereas no notable change was observed with collagen. The enhancements in anabolic signaling (mTORC1 and rpS6) along with elevated postprandial aminoacidemia provided support for these outcomes. The results indicated that an increase in protein consumption beyond the RDA facilitates improvements in MPS among older adults, although the quality of the protein remains a critical factor in addressing anabolic resistance (47).

A randomized controlled human model trial investigated the differential chronic impacts of whole protein/isolate animal-derived (whey) vs. plant-derived (pea) protein diets on MPS among both genders of middle-aged to older adults (ages 50–70, *n* = 27). Participants ingested a daily intake of 1.0 g/kg of protein over a duration of 10 days while engaging in unilateral resistance exercise training. The daily integrated myofibrillar protein synthesis was markedly elevated in the exercised leg in comparison to the non-exercised leg within both the whey (1.44 ± 0.26 vs. 1.29 ± 0.27%·day^−^^1^) and pea protein cohorts (1.50 ± 0.17 vs. 1.34 ± 0.21%·day^−^^1^), exhibiting no significant variances between the protein sources. Additional metrics, encompassing anabolic signaling, muscular strength, metabolic rate, and renal functionality, remained unchanged regardless of dietary intervention. Nonetheless, non-HDL cholesterol levels exhibited a reduction solely within the pea protein cohort (*P* = 0.014) (48).

A human model research aimed to formulate isolate and whole protein-enhanced restructured beef steaks specifically tailored for both genders of the geriatric population, utilizing plant-derived components such as PPI, rice protein, and lentil flour, along with the enzyme transglutaminase in a chronic evaluation framework. A total of 30 distinct formulations were meticulously evaluated to achieve an optimal balance between protein concentration and textural attributes. The maximum protein concentration, reaching as high as 28% in the thermally processed product, was realized through the strategic combination of pea, rice, and lentil protein sources. The textural characteristics were predominantly modulated by the presence of transglutaminase, whereas lentil flour contributed positively to the textural enhancements. A refined formulation comprising 2% transglutaminase, 8% pea protein, 9.35% rice protein, and 4% lentil flour demonstrated a reduction in hardness while enhancing the structural integrity. Nevertheless, consumer evaluations conducted with a cohort of 120 individuals aged over 65 indicated a preference for control products devoid of plant proteins, with seasoned formulations receiving the least favorable ratings (49).

Another randomized human model investigation examined the chronic effects of 13 days of supplementation with isolate pea protein, whey protein, and a placebo among both genders of physically active older adults (≥60 years, *n* = 47; final *n* = 15/group after attrition) to evaluate exercise-induced muscle damage (EIMD) subsequent to a 20–30 km walking session. Participants ingested 25 g/day of either whey protein, pea protein, or a placebo. Markers of muscle damage (CK, LDH), as well as muscle strength, muscle mass, and soreness, were assessed up to 72 h following the exercise intervention. Whey protein significantly mitigated the post-exercise elevation of creatine kinase at the 24-h mark when compared to pea protein and placebo (175 ± 90 vs. 300 ± 309 vs. 330 ± 165; *P* < 0.001). No statistically significant disparities were identified in relation to LDH, muscle strength, muscle mass, or soreness among the groups (50).

A 12-week chronic double-blind randomized controlled human model trial investigated the effects of whole protein whey, casein, and carbohydrate placebo supplementation in conjunction with supervised high-intensity resistance training among non-trained older males (62.5 ± 6.8 years, *n* = 36). All cohorts engaged in training sessions three times per week. Notable enhancements over time were documented in bench press strength, leg press strength, and fat-free mass across all participants. A significant interaction between group and time was identified for leg press strength, with whey (413.0 ± 108.7 kg) and casein (408.2 ± 130.8 kg) demonstrating a tendency toward superior gains in comparison to placebo (330.2 ± 85.2 kg), although not all observed differences achieved statistical significance. No further significant effects were noted regarding body composition or alternative strength parameters (51).

A self-regulated human model investigation examined the chronic implications of approximately 36 g/day of high-protein supplementation (whole protein casein plus whey) on muscle mass and gut microbiota in both genders of hospitalized elderly individuals (aged 60–90 years, *n* = 43) over a duration of 3 months, succeeded by a 3-month control interval. In male participants, the skeletal muscle mass index exhibited a statistically significant increase during the supplementation phase (from 6.0 to 6.3 kg/m^2^; *P* < 0.05) yet demonstrated a decline post-intervention, whereas no noteworthy changes were detected in female subjects. The composition and diversity of gut microbiota were influenced by protein consumption, with male subjects displaying an enrichment of advantageous bacterial species linked to muscle mass. Functional analyses indicated an elevation in microbial pathways associated with amino acid biosynthesis, which correlated with muscle-related taxa such as Blautia wexlerae. These results suggest that high-protein intake may facilitate muscle anabolism in older males, potentially through the modulation of gut microbial composition and metabolic activity, thereby reinforcing the concept of a gut–muscle axis in the context of age-related muscle degeneration (52).

In a separate randomized controlled human model trial investigated the chronic efficacy of fast-digesting whole protein whey vs. slow-digesting casein, in conjunction with mixed power training (MPT), on the enhancement of muscle function in older males (69 ± 7 years, *n* = 60). Participants were allocated to one of three groups: placebo + MPT (*n* = 19), whey protein + MPT (*n* = 21), or casein + MPT (*n* = 20), over a duration of 12 weeks. All intervention groups exhibited significant improvements in lean mass, muscle strength, muscle quality, and functional capacity subsequent to the training regimen. Nonetheless, no additional advantages were discerned with either protein supplementation in comparison to exercise alone. Serum metabolic markers and muscle characteristics exhibited analogous enhancements across all groups, with no significant differences identified between them. These results elucidated that when daily protein consumption exceeds the recommended dietary allowance, the incorporation of 30 g/day of either fast- or slow-digesting protein does not yield further improvements in training-induced muscle adaptations among older adults (53).

Another randomized clinical human model trial assessed the chronic effects of early enteral nutrition utilizing hydrolysate whey protein in comparison to whole protein casein among both genders of elderly patients suffering from ischemic stroke (median age 74 years, *n* = 31; completed *n* = 25) over a duration of 5 days. Participants were administered isocaloric and isonitrogenous nutritional formulas through nasogastric feeding. Mortality rates were comparable between the groups (33%). Nevertheless, the inflammatory and antioxidant responses exhibited notable differences: only the group receiving whey protein demonstrated a statistically significant reduction in interleukin-6 (IL-6; *P* = 0.02) alongside an elevation in glutathione levels (*P* = 0.03). Conversely, albumin concentrations were observed to decline exclusively within the casein group (*P* < 0.01). Between-group analyses substantiated the findings of lower IL-6 and elevated glutathione levels in the whey protein group when juxtaposed with the casein group (54).

A 16-week chronic randomized controlled human model trial examined the impact of a high-protein diet (HPD) enriched with either whole protein casein or soy protein in both genders of an elderly cohort in Singapore (*n* = 55). Participants adhered to either a wholesome dietary regimen alone (control, *n* = 19) or supplemented with 20 g/day of casein (HPD-CP, n = 18) or soy protein (HPD-SP, *n* = 18). The control group exhibited elevations in triglyceride levels and augmented composite cardiovascular risk indices. Conversely, both HPD groups preserved lipid profiles and cardiovascular risk parameters, with the soy protein group demonstrating a statistically significant decrease in total cholesterol levels. All groups experienced increases in CD34+ endothelial progenitor cells, whereas the soy protein group notably enhanced the angiogenic capacity of endothelial cells. No significant alterations were recorded in other vascular function metrics (55) (Table 5).

**Table 5:** Chronic effects of pea protein vs. dairy protein on muscle protein metabolism, amino acid availability, and functional outcomes in aging: a summary of clinical evidence of human studies.

Age/gender/ health status	Time horizon	Dose & duration	Form of protein/type of protein	Key outcomes	Findings	References
~72 ± 4 y/Males/Anabolic resistance	Chronic (>7 days)	50 g/day for 8 days (following 7 days control diet)	Isolate/Whey vs. Pea vs. Collagen above RDA baseline	Integrated MPS, anabolic signaling, aminoacidemia	Integrated MPS: ↑ whey = ↑ pea > ↔ collagen • mTORC1 signaling: ↑ whey = ↑ pea > ↔ collagen • Aminoacidemia: ↑ whey = ↑ pea > ↔ collagen	(47)
50–70 years/Both Genders/Healthy	Chronic (>7 days)	10 days; 1.0 g/kg/day protein + unilateral training	Whole protein/Isolate/ Whey-based vs. Pea-based diet + unilateral RET	iMyoPS, strength, metabolic markers	iMyoPS (trained leg): ↑ RET effect in both diets • Whey vs. pea: ↔ (no difference) • Strength: ↑ RET only • Metabolic rate: ↔ • Renal function: ↔ • Nitrogen balance: ↔ • Non-HDL cholesterol: ↓ pea diet only	(48)
>65 years/Both Genders/Consumer cohort	Chronic (>7 days)	Systematic prototype optimization matrix	Isolate & Whole protein/PPI, Rice protein, Lentil flour + Transglutaminase	Protein content, texture, consumer acceptability	Protein content: ↑ PPI/RP/LF formulation (up to 28%) • Binding strength: ↑ TG-dependent • Texture: ↑ LF improves softness • Hardness: ↓ optimal formulation • Consumer acceptability: ↓ plant-enriched steaks < control • Overall acceptability: ↓ reformulated products	(49)
≥60 years/Both Genders/Physically active	Chronic (>7 days)	13 days daily supplementation + acute walking	Isolate/Whey (25 g/day) vs. Pea (25 g/day) vs. Placebo	CK, LDH, muscle soreness, strength	CK (muscle damage marker): ↓ whey < pea = placebo • LDH: ↔ • Muscle strength: ↔ • Muscle soreness: ↔ • Skeletal muscle mass: ↔ • Overall protection vs. EIMD: ↑ whey only	(50)
~69 ± 7 y/Males/Healthy	Chronic (>7 days)	12 weeks; 30 g/day protein + power training 3 × /week	Whole protein/Whey vs. Casein vs. Placebo + power training	Muscle mass, strength, functional capacity	Muscle strength: ↑ all groups (training effect) • Lean mass: ↑ all groups • Functional capacity: ↑ all groups • Between groups: ↔ (no difference) • Protein type effect: ↔	(53)
~62.5 ± 6.8 y/Males/Untrained	Chronic (>7 days)	12 weeks; 20 g/day protein + resistance training 3 × /week	Whole protein/Whey vs. Casein vs. Carbohydrate placebo	Strength, FFM, body composition	1RM strength: ↑ all groups (training effect) • Leg press strength: ↑ WP ≈ casein > CHO (trend) • Fat-free mass: ↑ all groups (small) • Between whey vs. casein: ↔ • Overall sarcopenia outcome: ↑ whey = casein + RT	(51)
~74 y/Both Genders/Acute ischemic stroke	Chronic (>7 days)^*^	5 days early enteral feeding continuous matrix	Hydrolysate (Whey) vs. Whole protein (Casein enteral formula)	CRP, IL-6, glutathione, albumin	IL-6: ↓ whey < casein • CRP: ↓ whey < casein • Glutathione: ↑ whey • Albumin: ↓ casein group • Inflammation: ↓ whey > casein • Antioxidant defense: ↑ whey	(54)
60–90 y/Both Genders/Hospitalized	Chronic (>7 days)	3 months protein phase vs. 3 months control interval	Whole protein/Mixed whey + casein (~36 g/day)	Muscle mass, gut microbiota, microbial function	Muscle mass (males): ↑ protein phase, ↓ after withdrawal • Females: ↔ • Microbiota diversity: ↑ protein intake • Beneficial bacteria (*Blautia, Corynebacterium*): ↑ • Amino acid metabolism pathways: ↑ • Gut–muscle axis: ↑	(52)
Older adults/Both Genders/Healthy or At-risk	Chronic (>7 days)	16 weeks high-protein diet intervention	Whole protein/Casein vs. Soy protein within a high-protein template	Lipids, CVD risk, vascular function	Triglycerides: ↓ soy = casein vs. control ↑ • Total cholesterol: ↓ soy group • CVD risk score: ↓ HPD groups vs. control ↑ • Vascular function: ↔ • Endothelial progenitor cells: ↑ all groups (no major differences) • Overall cardiovascular protection: ↑ soy > casein	(55)

^*^Ref (26, 27) evaluates a continuous 5-day acute clinical enteral feeding model. While it sits just below the 7-day threshold, it constitutes continuous, multi-day therapeutic clinical exposure rather than single-dose postprandial kinetic tracking, aligning it methodologically with chronic adaptive markers.

AA, amino acids; ALAC, α-lactalbumin; AUC, area under the curve; BCAA, branched-chain amino acids; BM, body mass; CHO, carbohydrate; CK, creatine kinase; CRP, C-reactive protein; CVD, cardiovascular disease; EAA, essential amino acids; EIMD, exercise-induced muscle damage; FFM, fat-free mass; FSR, fractional synthetic rate; GLP-1, glucagon-like peptide-1; HDP/HPD, high-protein diet/hybrid dairy-plant proteins; iAUC, incremental area under the curve; IL-6, interleukin 6; iMyoPS, integrated myofibrillar protein synthesis; LDH, lactate dehydrogenase; LF, lentil flour; LPS, lipopolysaccharide; MCI, mild cognitive impairment; MPS, muscle protein synthesis; mTORC1, mammalian target of rapamycin complex 1; P4, hybrid dairy–plant protein blend; PE, pancreatic extract; PEA, pea protein; PPF, plant protein fiber products; PPI, pea protein isolate; RET, resistance exercise training; RMR, resting metabolic rate; RER, respiratory exchange ratio; RP, rice protein; SIRT1, sirtuin 1; SMC/PCg, supplementary motor cortex and posterior cingulate gyrus; SMP, soluble milk protein; TG, transglutaminase; TMP/TMC/TMF, total milk peptides/total milk casein/placebo; TRP, tryptophan; VAS, visual analog scale; WPC/WPI, whey protein concentrate/whey protein isolate; WPF, whey protein with fiber.

## 4 Mechanisms of action and clinical translation

### 4.1 Biological pathways

**Figure 1:**
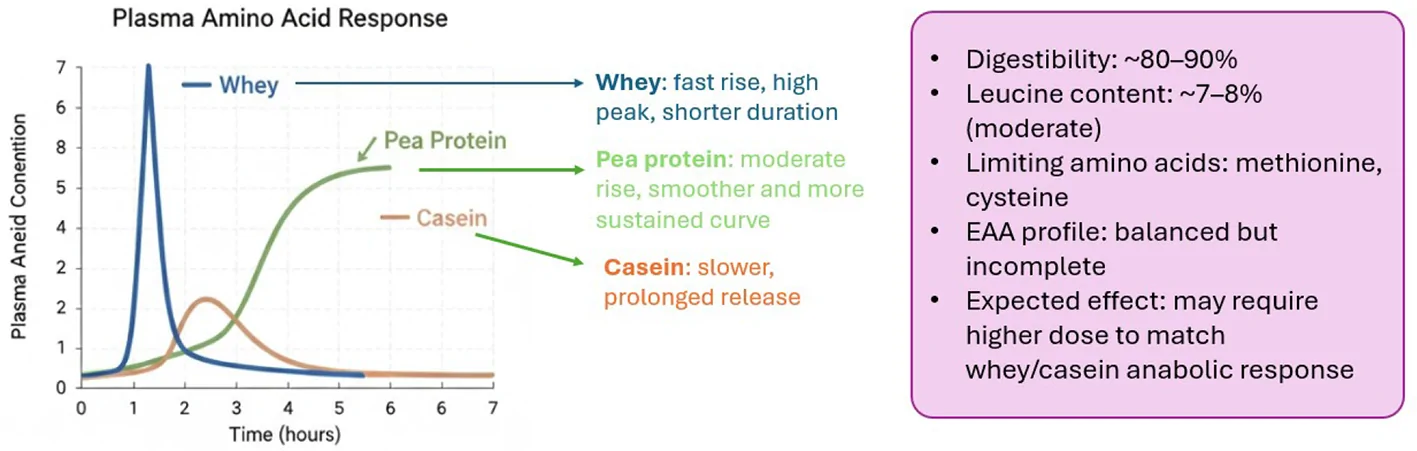
Comparative digestion kinetics and amino acid appearance of dietary proteins.

Leucine constitutes the primary anabolic initiator among EAAs, functioning as a pivotal modulator of the mTORC1 signaling cascade, which commences the process of MPS (56). Sufficient intake of leucine is particularly crucial for older individuals due to the phenomenon of anabolic resistance, which necessitates elevated thresholds to effectively stimulate MPS (57). Dairy-derived proteins, notably whey and α-lactalbumin, exhibit a higher concentration of leucine (~10%−12%) in contrast to pea protein (~7%−8%), leading to a more pronounced anabolic response (9, 58). Collectively, EAAs facilitate protein accretion; however, leucine serves as the “trigger,” whereas other EAAs function as substrates. Plant-based proteins frequently necessitate a greater overall intake or the incorporation of complementary amino acid sources to attain comparable anabolic efficacy as dairy proteins. Accordingly, the disparities in leucine concentration and the completeness of EAAs predominantly elucidate the enhanced MPS response observed with dairy proteins in comparison to plant-based alternatives in older populations (24, 56) (Figure 1).

Schematic representation of postprandial plasma amino acid responses following ingestion of whey, pea, and casein proteins. Whey protein exhibits rapid digestion and absorption, producing a sharp and early peak in plasma amino acid concentration with a shorter duration. Pea protein demonstrates intermediate digestion kinetics, characterized by a moderate increase and a more sustained amino acid release over time. In contrast, casein is digested more slowly, resulting in a delayed and prolonged amino acid appearance in circulation. Pea protein typically shows digestibility of approximately 80%−90%, contains moderate leucine levels (~7%−8%), and is relatively limited in sulfur-containing amino acids (methionine and cysteine). These compositional characteristics may contribute to a somewhat lower stimulation of muscle protein synthesis compared with rapidly digested dairy proteins, potentially requiring higher intake to achieve comparable anabolic responses (19, 20).

Postprandial aminoacidemia is influenced by the rate of protein digestion, the kinetics of gastric emptying, and the efficacy of amino acid transport mechanisms (59). Dairy proteins, especially rapid-digesting whey proteins such as α-lactalbumin, facilitate accelerated absorption dynamics, culminating in a pronounced and prompt elevation in plasma amino acid levels. In contrast, casein engenders a more gradual and prolonged release of amino acids due to the formation of gastric clots, thereby prolonging the availability of amino acids over an extended temporal framework. Consequently, this results in elevated peak aminoacidemia with dairy proteins when juxtaposed with plant-derived proteins, which generally exhibit more tempered and delayed responses. Nonetheless, certain investigations suggest that the total amino acid exposure (as represented by the AUC) following elevated dosages of pea protein can approximate that of dairy proteins, notwithstanding the comparatively lower peak concentrations. This observation implies that bioavailability may be analogous when dosages are equated. Strategies for protein blending, such as the amalgamation of pea protein with casein, can enhance both rapid and sustained amino acid provision, thereby augmenting anabolic efficiency in the geriatric population (19, 60, 61).

Through various investigations involving older individuals and aged animal models, the anabolic and metabolic impacts of pea protein, whey, casein, and various blended protein sources are predominantly influenced by discrepancies in postprandial aminoacidemia, particularly concerning the availability of leucine and the kinetics of digestion. Whey protein, characterized by rapid digestion, along with leucine-enhanced or blended proteins, elicits a more pronounced activation of mTORC1 signaling pathways and their downstream effectors (e.g., 4E-BP1, p70S6K), resulting in elevated or comparable MPS in comparison to plant-derived proteins. Plant proteins, including pea protein, have the potential to elicit analogous anabolic responses when administered in larger quantities, combined with complementary amino acids, or supplemented with leucine. Nonetheless, they frequently generate diminished peak responses in EAAs and insulin levels. Additionally, the source of protein influences gut microbiota composition, inflammatory responses, mitochondrial functionality, and signaling along the gut–brain axis, thereby affecting metabolic and neurocognitive outcomes. In summary, the quality of protein, its amino acid profile, and the rate of digestion collectively dictate the anabolic efficiency within aging tissues.

**Figure 2:**
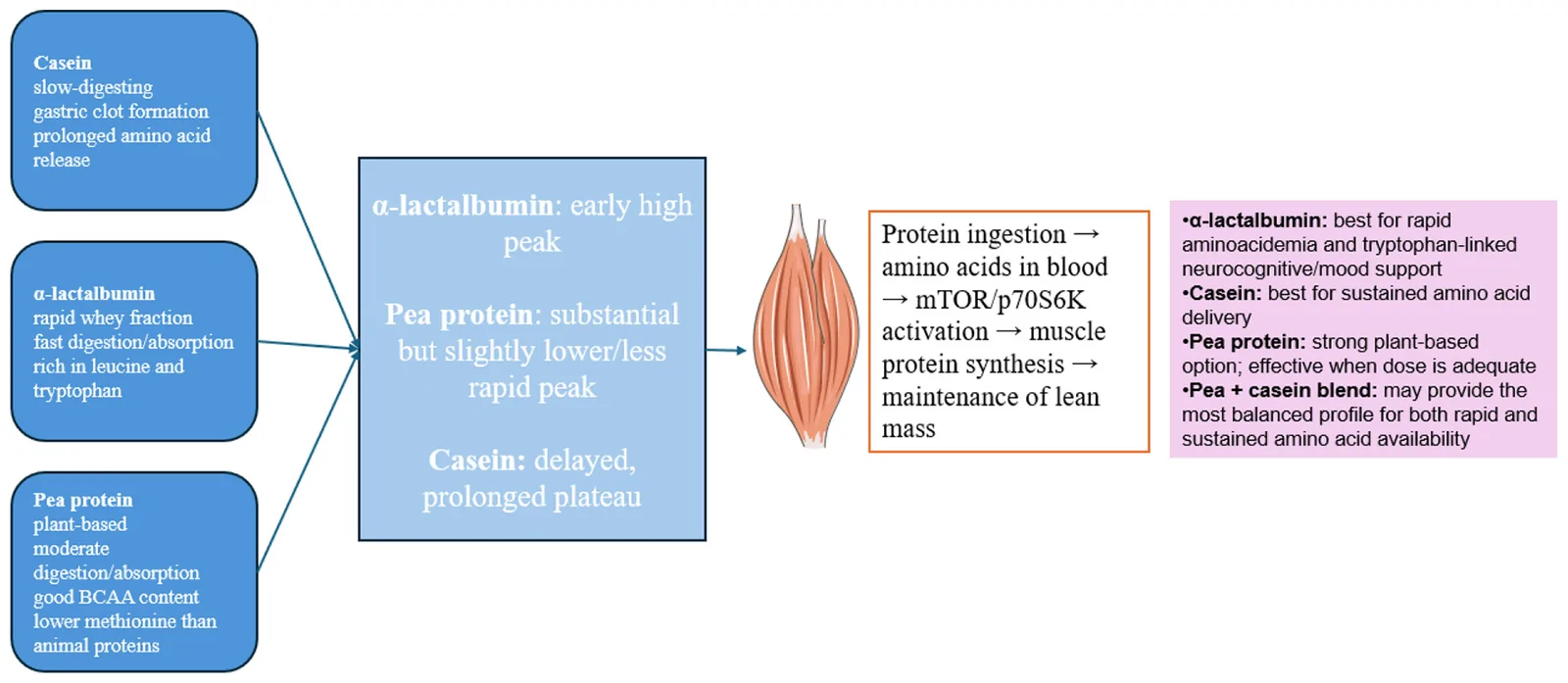
Comparative effects of pea protein, α-lactalbumin, and casein in older adults.

Across these investigations involving elderly individuals and aged animal models, the effects that depend on the protein source are predominantly influenced by variations in digestion kinetics, amino acid composition, and postprandial signaling mechanisms. Especially rapid-digesting whey proteins such as α-lactalbumin consistently elicit elevated levels of leucinemia, enhanced activation of mTORC1 signaling pathways, and augmented MPS compared to intact casein or the majority of plant-derived proteins. Although pea and other plant proteins can achieve similar anabolic responses when administered in increased amounts, combined, or supplemented with leucine, they typically result in lower peak levels of EAAs and insulin responses. In addition to muscle-related effects, the source of protein influences the composition of gut microbiota, pathways related to inflammation and oxidative stress, mitochondrial functionality, and signaling along the gut–brain axis, thereby affecting metabolic, vascular, and neurocognitive outcomes. Casein offers a slower yet sustained release of amino acids, which aids in maintaining net protein balance, while hydrolysates enhance kinetics without necessarily improving anabolic efficiency. In summary, the quality of protein, the timing of its intake, and the effects of its matrix collectively shape the integrated physiological responses related to aging (Figure 2).

Pea protein provides a plant-based alternative with good digestibility and BCAA content, α-lactalbumin produces rapid aminoacidemia and may support mood-related outcomes via its tryptophan content, and casein provides slower, prolonged amino acid release. Together, these proteins differ in their effects on postprandial aminoacidemia, muscle protein synthesis, appetite/metabolic responses, gut microbiota, and neurocognitive function. Protein blending may combine the rapid anabolic advantages of α-lactalbumin with the sustained release properties of casein, while pea protein remains a viable option when adequately dosed or supplemented (19, 20, 27).

### 4.2 Functional domain synthesis

#### 4.2.1 Aminoacidemia and amino acid bioavailability

In various human clinical trials, dairy-derived proteins consistently elicited a more rapid and significant postprandial aminoacidemia when juxtaposed with pea protein, a phenomenon predominantly attributable to elevated concentrations of EAAs, most notably Leucine. Whey protein exhibited enhanced peak levels of leucinemia and exhibited accelerated absorption kinetics, in contrast, pea protein produced analogous cumulative amino acid exposure over temporal assessments across multiple investigations. Utilization of blended methodologies (for instance, casein–pea combinations) partially mitigated the deficiencies in methionine and leucine content inherent to pea protein, thereby yielding intermediate amino acid profiles. Notably, when the leucine concentration was equivalently matched or augmented, plant-based proteins attained comparable systemic amino acid bioavailability, underscoring the primacy of amino acid composition over protein origin as a pivotal determinant.

#### 4.2.2 MPS and anabolic resistance

Both clinical and preclinical investigations suggest that pea protein possesses the capability to enhance MPS to levels that are comparable to those seen with dairy proteins, provided that appropriate dosing or leucine supplementation is utilized. Nevertheless, under typical physiological conditions, whey protein continues to demonstrate superior efficacy in the acute activation of anabolic signaling cascades, such as the mTOR pathway. Casein, characterized by its gradual digestion process, facilitates an extended release of amino acids; however, it exhibits a diminished acute MPS response. Importantly, findings from long-term studies have indicated negligible differences among various protein sources in terms of integrated MPS, implying that the overall protein intake may be more critical than the specific source in fostering chronic physiological adaptations (47, 48).

#### 4.2.3 Appetite regulation and energy metabolism

Evidence derived from randomized controlled trials indicates an absence of consistent discrepancies between pea and dairy protein sources concerning appetite-related hormones (e.g., ghrelin, GLP-1), perceived satiety, or overall energy consumption. Both protein sources exerted analogous effects on postprandial energy expenditure as well as substrate oxidation. Age-associated reductions in metabolic rate were noted irrespective of the protein source, suggesting that the type of protein has a limited influence on short-term appetite and energy homeostasis.

#### 4.2.4 Gut microbiota and metabolic health

Emerging research indicates that the administration of protein supplements, irrespective of their origin, has the potential to influence the composition of gut microbiota and metabolic pathways in geriatric populations. However, plant-derived proteins, such as pea protein, may provide supplementary advantages by promoting fiber-related effects and fostering greater microbial diversity (62). These alterations in microbiota composition have been associated with enhanced amino acid metabolism and improved muscle-related outcomes, thereby reinforcing the hypothesis of a gut–muscle axis. Nonetheless, empirical comparisons between pea protein and α-lactalbumin in this specific area of study remain scarce.

#### 4.2.5 Inflammation and oxidative stress

Dairy-derived proteins, with particular emphasis on α-lactalbumin, exhibit compelling evidence for their anti-inflammatory and antioxidant properties in preclinical investigations, which encompass decreases in markers indicative of oxidative stress and the modulation of age-related biological pathways such as SIRT1 (36). Conversely, plant-derived proteins display neutral to modest influences on inflammatory markers in human research, with certain advantages becoming apparent when synergistically combined with additional bioactive compounds (e.g., inulin) (30). In summary, although the preferential anti-inflammatory efficacy of dairy proteins is implied, it remains to be conclusively validated in human populations.

#### 4.2.6 Neurocognitive function and mood

α-Lactalbumin exerts a unique influence on neurocognitive outcomes through its elevated tryptophan concentration and its capacity to modulate tryptophan metabolism. Empirical investigations involving older adults, including those exhibiting mild cognitive impairment, reveal an enhancement in tryptophan availability and its subsequent metabolites subsequent to the consumption of α-lactalbumin (43). Nonetheless, these biochemical alterations did not consistently result in immediate enhancements in mood or cognitive functioning. Conversely, the absence of any established direct neurocognitive effects stemming from pea protein signifies a significant lacuna in the extant research.

#### 4.2.7 Functional outcomes and muscle performance

Intervention studies suggest that both plant-based and dairy-derived proteins can enhance muscle strength, lean body mass, and functional capacity when synergistically utilized with resistance training. Nevertheless, whey protein exhibits distinct advantages in mitigating EIMD, while pea protein seems to be comparatively less effective within this framework (50). Casein reveals similar long-term effects on muscle function but lacks robust evidence to support superior performance outcomes (51, 53).

#### 4.2.8 Overall interpretation

Collectively, the evidence suggests that:

- Pea protein is effective and comparable under optimized conditions (adequate dose, leucine enrichment, or blending strategies).
- Dairy proteins (especially whey and α-lactalbumin) exhibit superior acute anabolic, anti-inflammatory, and neurochemical effects.
- Casein provides sustained amino acid delivery but lower acute anabolic stimulation.

Collectively, the protein source matters in acute physiology, whereas total protein intake and dietary strategy dominate long-term outcomes in aging populations.

### 4.3 Clinical implications

The clinical implications derived from the extant evidence suggest that the protein intake of older adults ought to be tailored to individual needs rather than prescribed uniformly, as the physiological response to dietary protein is significantly modulated by factors such as age-related anabolic resistance, baseline health status, and functional priorities. In geriatric populations, elevated protein requirements may be imperative to maintain MPS and mitigate sarcopenia, especially in those exhibiting diminished physical activity, acute illnesses, or chronic inflammatory conditions. Conversely, individuals with cognitive vulnerabilities, such as those diagnosed with mild cognitive impairment, may derive benefits from protein sources that positively affect neurotransmitter pathways, including the metabolism of tryptophan, thereby indicating that the selection of protein may encompass considerations beyond musculoskeletal outcomes to also include aspects of brain health. Furthermore, functional objectives are pivotal in the optimization of dietary strategies; whereas certain individuals may emphasize the preservation of muscle mass and physical performance, others might prioritize metabolic health or the maintenance of neurocognitive function, necessitating a more precise nutritional approach. From a comprehensive nutritional standpoint, plant-derived proteins such as pea protein present distinct benefits regarding ecological sustainability and prolonged dietary compliance. Conversely, proteins sourced from animals, especially dairy proteins like casein and α-lactalbumin (as a subgroup of whey protein), typically exhibit enhanced acute anabolic effectiveness owing to their accelerated digestion rates and elevated leucine concentrations. Consequently, a practical approach might not depend solely on a singular protein source but rather on a flexible, individualized strategy that harmonizes efficacy, metabolic requirements, and considerations for sustainability.

Furthermore, tailoring these personalized strategies requires a careful evaluation of potential side effects inherent to specific protein extractions, which ultimately restricts the broad generalizability of a uniform dietary model. For example, plant-derived alternatives such as pea protein isolates possess moderate to high concentrations of endogenous purines. While plant-based purine sources are generally associated with a lower epidemiological risk of metabolic complications than animal-derived purines, their concentrated intake may still present clinical risks by exacerbating hyperuricemia or triggering acute inflammatory flares in patients susceptible to gout (63, 64). For this reason, clinical recommendations cannot be uniformly applied across all geriatric cohorts and must be heavily adapted to individual metabolic baselines.

### 4.4 Methodological limitations and heterogeneity

#### 4.4.1 Appraisal of evidence on pea protein anabolic and metabolic effects in aging

Current literature juxtaposing pea protein with dairy proteins in geriatric populations and aged models is constrained by diminutive sample sizes (typically *n* = 9–47 in human subjects) and abbreviated intervention durations (acute 3–5 h postprandial assessments or 10–16 day trials), thereby diminishing external validity regarding long-term muscular outcomes. Numerous investigations are characterized by crossover or single-blind methodologies with incomplete blinding, thereby heightening the susceptibility to performance and expectation biases. Animal studies (n ≈ 30–40) yield mechanistic insights; however, they possess limited translational applicability due to interspecies variances in protein metabolism. The heterogeneity of outcomes (MPS, amino acid levels, hormonal responses, microbiota) along with inconsistent protein dosing further obfuscates comparative analyses. A number of trials are underpinned by industry sponsorship, which introduces the potential for funding biases. Furthermore, numerous studies are deficient in standardized resistance exercise control, which constitutes a significant confounder in MPS investigations. In summary, whilst whey protein consistently elicits higher leucinemic responses, the evidence supporting the equivalence of plant-derived proteins remains nascent and contextually contingent.

#### 4.4.2 Methodological limitations in studies comparing plant and dairy protein effects on aging outcomes

The extant evidence framework is constrained by predominantly small sample sizes (*n* ≈ 9–60 in human investigations; ~30–48 in animal experiments) and brief intervention durations that range from acute postprandial evaluations (3–5 h) to short-term interventions (5 days−12 weeks), with only a limited number extending to a duration of 6 months. Numerous studies adopt crossover or single-/double-blind methodologies; however, several fail to implement complete blinding of dietary interventions, thus introducing potential biases related to performance and expectations. The variability in protein dosage, amino acid matching, and co-interventions (e.g., resistance training, dietary fiber, or micronutrients) constrains the comparability across various studies. Animal research offers mechanistic insights yet possesses limited translational relevance to human subjects. A number of trials are financed by industry sponsors, which raises concerns regarding potential sponsorship bias. Furthermore, numerous studies do not account for habitual dietary protein consumption or physical activity levels, thereby confounding outcomes related to MPS and metabolic health. In summary, the evidence supports whey protein's superiority for acute MPS; however, the long-term clinical superiority remains ambiguous.

### 4.5 Limitations of current evidence

Notwithstanding the expanding corpus of research juxtaposing pea and dairy proteins within aging demographics, several critical limitations must be recognized when assessing the extant literature. Primarily, the preponderance of existing studies constitutes short-term interventions, frequently spanning from acute postprandial assessments to a few weeks of supplementation. Such methodological frameworks are inadequate for elucidating long-term adaptations in muscle mass, functional capacity, neurocognitive performance, or metabolic health, thereby constraining the translational applicability of the findings to chronic aging-related phenomena such as sarcopenia or cognitive decline. Furthermore, there exists substantial heterogeneity in study design, encompassing variations in protein dosage, timing of administration, co-ingested nutrients, physical activity status, and participant characteristics. This variability renders direct comparisons across studies problematic and amplifies the risk of drawing inconsistent conclusions regarding the relative efficacy of plant-based vs. animal-based proteins. Third, while α-lactalbumin has demonstrated encouraging biological impacts, especially in the modulation of tryptophan metabolism and inflammatory mechanisms, the existing human evidence is still scarce and predominantly confined to studies of limited scale or short duration. There is an absence of long-term randomized controlled trials that assess its effects on functional or clinical outcomes. Fourth, a significant deficiency exists in the body of research concerning the neurocognitive implications of pea protein, notwithstanding the emerging data that associates amino acid metabolism with cerebral health in the context of aging. This indicates a critical area that has yet to be thoroughly investigated, particularly in light of the growing interest in plant-based dietary practices and cognitive aging. Ultimately, a significant portion of mechanistic understanding is obtained from both animal studies and *in vitro* experiments, encompassing aged rodent models and senescence-accelerated mouse strains. Although these experimental models yield important mechanistic insights, their relevance to human physiological processes is inherently constrained. Collectively, these limitations underscore the imperative for meticulously designed, longitudinal, and standardized clinical trials to more accurately elucidate the influence of protein sources on healthy aging.

While this review establishes separate, predefined eligibility streams for human and animal evidence to maximize structural clarity, it is bound by the inherent methodological constraints of a narrative framework. Consequently, we did not perform a formal, standardized evaluation of the risk of bias or methodological quality (such as Cochrane or SYRCLE profiling) for the included literature. We recognize that independent, comprehensive systematic reviews and meta-analyses represent the absolute gold standard for establishing clinical practice guidelines and shaping public health policies. The synthesis provided here is intended to serve as an integrative, mechanistic roadmap to highlight system-level axes and guide future target-driven clinical food science trials, rather than to serve as a rigid tool for clinical diagnostic policy.

## 5 Sources of heterogeneity and methodological limitations

### 5.1 Study design and time horizon divergence

A primary source of methodological heterogeneity stems from the steep divergence between acute and chronic experimental time horizons. Preclinical and clinical evidence is sharply split between acute postprandial kinetic tracking (less than 24 h) and chronic adaptive feeding frameworks (greater than 7 days). While acute human and animal models are ideal for evaluating immediate intestinal amino acid transporter saturation, peak peripheral hyperaminoacidemia, and short-term signaling cascades, these temporary metabolic spikes do not consistently mirror long-term phenotypic changes. Consequently, chronic human and animal designs evaluating prolonged exposure are vital to determine if transient kinetic variations translate into lasting adjustments in lean mass or structural tissue morphology.

### 5.2 Protein source, matrix, and administration heterogeneity

Considerable variation exists regarding the physical processing forms and delivery matrices of the investigated proteins. Studies fluctuate between highly purified isolates (e.g., whey protein isolate [WPI] or pea protein isolate [PPI]) and less processed concentrates or whole food matrices. These structural variations modify digestion kinetics, as isolates lack the matrix-bound components that delay gastric emptying. Furthermore, the operational context of protein delivery introduces confounding variables: several protocols evaluate protein administration in tandem with mechanical stimulus (pre- or post-exercise physical activity), which fundamentally alters muscle sensitivity to hyperaminoacidemia, whereas other designs evaluate sedentary, resting baselines. Finally, the physical route of administration differs significantly, moving from highly controlled, stress-inducing involuntary oral gavage protocols in animal lines to voluntary, palatable beverage ingestions in clinical human cohorts.

### 5.3 Outcome-specific limitations: muscle protein synthesis vs. gut microbiota

The physiological systems evaluated in this review suffer from distinct, outcome-specific evidentiary limitations:

- **MPS:** The vast majority of metabolic data focuses strictly on acute fractional synthetic rates measured over a window of a few hours. These short-term skeletal muscle synthesis spikes lack validation from long-term, longitudinal functional metrics such as grip strength, real-world physical performance, or structural cross-sectional mass.
- **Gut microbiota dynamics:** Conversely, data regarding microbiome shifts remain heavily weighted toward animal models. Preclinical rodent models permit aggressive tissue harvesting and strict environmental isolation, but their translational value remains unclear due to stark species-specific variations in baseline enterotypes, anatomical fermentation sites (cecal vs. colonic), and highly controlled experimental diets that fail to reflect the erratic, poly-pharmaceutical reality of aging human populations.

### 5.4 Biological blunting and anabolic resistance

When interpreting clinical translation, a major confounding biological variable is the presence of age-related anabolic resistance. Aging skeletal muscle exhibits a blunted sensitivity to both hyperaminoacidemia and low-dose intracellular leucine signaling. Because many foundational mechanistic studies utilize young, highly responsive biological baselines, their optimistic anabolic outcomes cannot be uniformly projected onto fragile older cohorts. Older populations require a substantially higher absolute per-meal per-dose leucine threshold to trigger comparable muscle protein synthetic responses, rendering uniform, cross-generational comparisons highly flawed.

### 5.5 Demographic and model discrepancies

Finally, cross-study generalizability is constrained by fundamental demographic and model mismatches across the literature:

- **Age and sex profile:** Compounding populations across wide developmental bands (young vs. old cohorts) obscures the unique metabolic needs of advanced age. Furthermore, existing human datasets are heavily male-dominated or report combined outcomes without sex-disaggregated analyses, leaving potential sex-dependent variations in muscle mass maintenance or splanchnic extraction in aging females entirely unclear.
- **Preclinical strain profiles:** In animal models, genetic background introduces subtle physiological variances; for example, Sprague-Dawley rats show a higher propensity for metabolic drifting and weight gain compared to the leaner, more immunologically stable Wistar rat strain.
- **Health and metabolic status:** Lastly, a distinct translational gap remains between highly standardized, disease-free preclinical animal strains and clinically complex, multi-morbid, or frail aging human populations whose underlying systemic inflammation may alter protein processing.

## 6 Future directions and conclusions

While the existing literature provides essential foundational knowledge regarding acute postprandial kinetics, translating these findings into robust, long-term clinical protocols requires a paradigm shift in study design. Future clinical food science trials must look beyond immediate biochemical surrogates, such as transient blood aminoacidemia or short-term muscle fractional synthetic rates, and capture hard, patient-centered clinical endpoints. Specifically, longitudinal, multi-center randomized controlled trials (RCTs) are urgently needed to track the long-term impact of targeted protein interventions on health-related quality of life (HRQoL) and all-cause hospitalization rates in the geriatric population. Evaluating healthcare utilization parameters, such as the frequency and duration of hospital admissions or the rate of frailty-related falls, will provide the definitive clinical and economic justification required to integrate specialized protein matrices into standard geriatric care guidelines.

Most notably, this review highlights a glaring gap in head-to-head comparative literature: to date, no clinical trial has directly contrasted the acute or chronic physiological impacts of pea protein isolate against pure α-lactalbumin fractions. Given that α-lactalbumin represents the premier rapid-digesting dairy benchmark for tryptophan delivery and neurocognitive signaling, while pea protein represents the primary expanding frontier for sustainable, leucine-rich plant alternatives, directly pairing these two matrices is a critical next step. Future research should prioritize large-scale, head-to-head human interventions comparing pea protein vs. α-lactalbumin across balanced gender cohorts. These trials should concurrently track the system-level axis proposed in this review, linking long-term muscle mass maintenance, gut microbiome stability, and neurocognitive performance, to definitively establish optimal, personalized nutritional strategies for diverse, vulnerable aging populations.

This narrative review synthesizes the comparative physiological impacts of plant-derived pea protein vs. dairy-derived casein and α-lactalbumin across the gut–brain–muscle axes in aging. Pea protein isolates demonstrate promising potential as sustainable alternatives to traditional dairy configurations. However, based on the available evidence, drawing definitive causal conclusions regarding their long-term clinical equivalence appears to be premature. The current body of evidence is highly constrained by prominent methodological limitations, including a predominance of acute (< 24 h) exposure frameworks, small sample sizes, and a distinct lack of long-term, head-to-head clinical randomized controlled trials (RCTs). Furthermore, because this review evaluates preclinical animal models and human clinical trials concurrently, direct cross-species translation of underlying metabolic mechanisms is limited by inherent physiological discrepancies. The generalizability of the current literature is also restricted by significant demographic and operational factors. High study heterogeneity regarding intervention protocols, protein matrices, and measurement tools complicates direct comparisons. Crucially, because existing cohorts are predominantly male-dominated or report combined outcomes without sex-disaggregated analyses, the clinical efficacy and safety profiles of these protein sources specifically in aging females remain unclear. Ultimately, while both protein sources exhibit distinct functional advantages, definitive dietary recommendations must await robust, well-powered, and long-term longitudinal clinical interventions that accommodate individual metabolic baselines and diverse gender cohorts.
